# Role of spinal cord alpha-amino-3-hydroxy-5-methyl-4-isoxazolepropionic acid receptors in complete Freund's adjuvant-induced inflammatory pain

**DOI:** 10.1186/1744-8069-4-67

**Published:** 2008-12-30

**Authors:** Jang-Su Park, Myron Yaster, Xiaowei Guan, Ji-Tian Xu, Ming-Hung Shih, Yun Guan, Srinivasa N Raja, Yuan-Xiang Tao

**Affiliations:** 1 Department of Anesthesiology and Critical Care Medicine, Johns Hopkins University School of Medicine, Baltimore, Maryland 21205, USA; 2 Department of Anesthesiology and Pain Medicine, Ilsan Paik Hospital, Inje University, Koyang 411706, South Korea; 3 Department of Anesthesiology, Chang Gung Memorial Hospital, Chia-Yi School, Chang Gung Institute of Technology, Chia-Yi, Taiwan 613, Republic of China

## Abstract

Spinal cord α-amino-3-hydroxy-5-methyl-4-isoxazolepropionic acid receptors (AMPARs) mediate acute spinal processing of nociceptive and non-nociceptive information, but whether and how their activation contributes to the central sensitization that underlies persistent inflammatory pain are still unclear. Here, we examined the role of spinal AMPARs in the development and maintenance of complete Freund's adjuvant (CFA)-induced persistent inflammatory pain. Intrathecal application of two selective non-competitive AMPAR antagonists, CFM-2 (25 and 50 μg) and GYKI 52466 (50 μg), significantly attenuated mechanical and thermal hypersensitivities on the ipsilateral hind paw at 2 and 24 h post-CFA injection. Neither CFM-2 nor GYKI 52466 affected the contralateral basal responses to thermal and mechanical stimuli. Locomotor activity was not altered in any of the drug-treated animals. CFA-induced inflammation did not change total expression or distribution of AMPAR subunits GluR1 and GluR2 in dorsal horn but did alter their subcellular distribution. The amount of GluR2 was markedly increased in the crude cytosolic fraction and decreased in the crude membrane fraction from the ipsilateral L_4–5 _dorsal horn at 24 h (but not at 2 h) post-CFA injection. Conversely, the level of GluR1 was significantly decreased in the crude cytosolic fraction and increased in the crude membrane fraction from the ipsilateral L_4–5 _dorsal horn at 24 h (but not at 2 h) post-CFA injection. These findings suggest that spinal AMPARs might participate in the central spinal mechanism of persistent inflammatory pain.

## Background

The α-amino-3-hydroxy-5-methyl-4-isoxazolepropionic acid (AMPA)-type ionotropic glutamate receptors (AMPARs) mediate most fast excitatory synaptic transmissions and play a critical role in synaptic plasticity in the mammalian central nervous system [1,2]. AMPARs are tetramers that comprise a combination of four subunits termed GluR1-4 [3]. Changes in postsynaptic membrane trafficking or in synaptic targeting of these AMPAR subunits alter synaptic strength and have been recognized as a central mechanism underlying various forms of synaptic plasticity [1,2].

Spinal central sensitization, a specific form of synaptic plasticity, is a mechanism that underlies the development and maintenance of pain hypersensitivity after peripheral inflammation [4,5]. In addition to mediating acute spinal processing of nociceptive and non-nociceptive information, the activation of spinal AMPA/kainate receptors might contribute to spinal central sensitization under inflammation-induced persistent pain conditions. Intrathecal pretreatment with AMPA/kainate receptor antagonists was shown to markedly reduce thermal injury-induced mechanical tactile allodynia, second-phase formalin-induced nociceptive behaviors, and carrageenan-induced thermal and mechanical hypersensitivities [6,7]. Because these antagonists are not highly selective for AMPARs, it is still unclear whether spinal AMPARs play a critical role in persistent inflammatory pain. In addition, these AMPA/kainate receptor antagonists also produce unwanted side effects [8], which limit their therapeutic potential in persistent pain.

Recent evidence suggests that peripheral inflammatory insults might regulate synaptic trafficking of AMPAR subunits in spinal cord. Capsaicin-induced acute visceral inflammatory insult rapidly increased the amount of GluR1 protein, but not GluR2 or GluR3 proteins, in the spinal cord membrane fraction and correspondingly decreased the level of GluR1 in the cytosolic fraction, without affecting total GluR1 or GluR2 protein expression in spinal cord [9]. The level of postsynaptic GluR1, but not GluR2 or GluR3, at lamina II nonpeptidergic C-fiber synapses was increased during capsaicin-induced acute inflammation [10]. Complete Freund's adjuvant (CFA)-induced persistent inflammation significantly elevates the amount of GluR1 in the postsynaptic density fraction from spinal cord [11]. This finding indicates that GluR1 could be recruited to the plasma membrane of spinal cord neurons by persistent noxious inflammation. A previous study reported that CFA-induced persistent inflammation increased expression of GluR1 and GluR2 mRNA and the density of total GluR1 and GluR2 immunohistochemical staining in dorsal horn [12], suggesting that the levels of GluR1 and GluR2 may be increased in both plasma membrane and cytosolic fractions of dorsal horn neurons after CFA injection. Thus, it is still unclear whether CFA-induced persistent inflammation, like capsaicin-induced acute inflammatory insult, leads to changes in synaptic trafficking of AMPAR subunits in dorsal horn neurons.

Here, we first characterized the role of AMPARs in CFA-induced persistent inflammatory pain in rats using two highly selective non-competitive AMPAR antagonists, 1-(4'-aminophenyl)-3,5-dihydro-7,8-dimethoxy-4*H*-2,3-benzodiazepin-4-one (CFM-2) and 4-(methyl-9*H*-1,3-dioxolo [4,5-h][2,3]benzodiazepin-5-yl)-benzenamine hydrochloride (GYKI 52466) [13-15]. We then examined whether CFA-induced peripheral inflammation altered expression and distribution of total GluR1 and GluR2 proteins in dorsal horn. Finally, we determined whether the amounts of GluR1 and GluR2 proteins were changed in crude plasma membrane and cytosolic fractions from dorsal horn during CFA-induced inflammatory pain conditions.

## Materials and methods

### Animal preparation

Male Sprague-Dawley rats (250–300 g) were housed in cages on a standard 12:12 h light/dark cycle. Water and food were available ad libitum until rats were transported to the laboratory approximately 1 h before experiments. The animals were used in accordance with protocols that were approved by the Animal Care and Use Committee at the Johns Hopkins University and were consistent with the ethical guidelines of the National Institutes of Health and the International Association for the Study of Pain. All efforts were made to minimize animal suffering and to reduce the number of animals used.

Intrathecal catheters were implanted into animals under isoflurane anesthesia. A polyethylene (PE-10) tube was inserted into the subarachnoid space at the rostral level of the spinal cord lumbar enlargement segment through an incision at the atlanto-occipital membrane according to the method described previously [16,17]. The animals were allowed to recover for 5–10 days before being used experimentally. Rats showing any neurologic deficits postoperatively were discarded from the study. The position of the PE-10 catheter was confirmed in each animal after behavioral testing.

### Experimental drugs

CFA was purchased from Sigma Chemical Co. (St. Louis, MO). 6-cyano-7-nitroquinoxaline-2,3-dione (CNQX, a competitive AMPA/kainate receptor antagonist), CFM-2 (a selective non-competitive AMPAR antagonist), and GYKI 52466 (a selective non-competitive AMPAR antagonist) were purchased from Tocris Bioscience (Ellisville, MO). CNQX was dissolved in 0.9% physiologic saline, whereas CFM-2 and GYKI 52466 were dissolved in 10% dimethylsulfoxide (DMSO).

### CFA-induced persistent inflammatory pain model

To induce persistent inflammatory pain, the rats were placed under isoflurane anesthesia, and 100 μl of CFA (1 mg/ml *Mycobacterium tuberculosis*) solution was injected into the plantar side of one hind paw. Our previous studies showed that significant CFA-induced thermal and mechanical pain hypersensitivities appeared at 2 h, reached a peak level between 6 and 24 h, and were maintained for at least 72 h [17]. Therefore, we chose 2 h and 24 h post-CFA injection to represent the development and maintenance phases of CFA-induced persistent inflammatory pain for the pharmacologic and biochemical studies.

To examine the role of spinal cord AMPARs in persistent inflammatory pain, two selective competitive AMPAR antagonists, CFM-2 (5, 25, or 50 μg/10 μl) and GYKI 52466 (50 μg/10 μl) were injected intrathecally followed by 10 μl of saline to flush the catheter (total volume of the catheter: 10 μl) at 2 h or 24 h post-CFA injection. To compare the effects of selective and non-selective AMPAR antagonists on persistent inflammatory pain, a competitive AMPA/kainate receptor antagonist, CNQX (6.1 μg/10 μl), was given according to the same protocol. Saline and 10% DMSO were used as controls. The behavioral tests described below were performed 1 day before CFA injection (baseline) and at 20 min after drug administration. Separate groups of rats were used for the 2-h and 24-h behavioral tests. The antagonist doses used were based on data from previous studies [18-21] and our pilot work. The experimenters were blinded to the treatment groups.

### Behavior testing

To measure paw withdrawal response to noxious heat stimuli, each animal was placed in a Plexiglas chamber on a glass plate located above a light box. Radiant heat from a Model 336 Analgesic Meter (IITC, Inc./Life Science Instruments, Woodland Hills, CA) was applied by aiming a beam of light through a hole in the light box through the glass plate to the middle of the plantar surface of each hind paw. When the animal lifted its foot, the light beam was turned off. The length of time between the start of the light beam and the foot lift was defined as the paw withdrawal latency. Each trial was repeated five times at 5-min intervals for each paw. A cut-off time of 20 s was used to avoid paw tissue damage.

To measure paw withdrawal response to repeated mechanical stimuli, each animal was placed in a Plexiglas chamber on an elevated mesh screen. A single trial of mechanical stimuli consisted of eight applications of a calibrated von Frey filament (8.01 mN, Stoelting Co., Wood Dale, IL) within a 2–3-s period. Each trial was repeated 10 times at 3-min intervals on each hind paw. The occurrence of hind paw withdrawal in each of these 10 trials was expressed as a percent response frequency, and this percentage was used as an indication of the amount of hind paw withdrawal.

### Locomotor function testing

To examine whether the antagonists used in behavioral testing affected the locomotor function, three reflexes (placing, grasping, and righting) were tested as described previously [16,22]. In brief, the naïve animals received a 10-μl intrathecal injection of vehicle (saline or 10% DMSO), CNQX (6.1 μg/10 μl), CFM-2 (50 μg/10 μl), or GYKI 52466 (50 μg/10 μl). Twenty minutes later, the following tests were performed with the experimenter blind to drug treatment: (1) Placing reflex: The experimenter held the rat with hind limbs slightly lower than the forelimbs and brought the dorsal surfaces of the hind paws into contact with the edge of a table. The experimenter recorded whether the hind paws were placed on the table surface reflexively; (2) Grasping reflex: The experimenter placed the rats on a wire grid and recorded whether the hind paws grasped the wire on contact; (3) Righting reflex: The experimenter placed the rat's back on a flat surface and noted whether it immediately assumed the normal upright position. Scores for placing, grasping, and righting reflexes were based on counts of each normal reflex exhibited in five trials. In addition, the rat's general behaviors, including spontaneous activity (e.g. walking and running), were observed.

### Western blot analysis

Expression of GluR1 and GluR2 proteins in the total soluble fraction, crude membrane fraction, and crude cytosolic fraction was examined. In brief, the animals were sacrificed by decapitation at 2 and 24 h after injection of saline (100 μl) or CFA into the plantar side of a hind paw. Naïve animals were used as controls. The L_4–5 _spinal cord ipsilateral and contralateral to CFA or saline injection was removed. The dorsal part of the spinal cord was separated from the ventral part and collected. Total soluble fraction, crude membrane fraction, and crude cytosolic fraction were prepared as described below. The samples were heated for 5 min at 99°C and then loaded onto 4% stacking/7.5% separating SDS-polyacrylamide gels. The proteins were electrophoretically transferred onto nitrocellulose membrane. The blotting membrane was blocked with 3% non-fat dry milk for 1 h and incubated overnight at 4°C with rabbit anti-GluR1 (1:200; Upstate/CHEMICON, Temecula, CA), rabbit anti-GluR2 (1:500; Upstate/CHEMICON), rabbit anti-*N*-cadherin (1:1,000; BD Biosciences, Palo Alto, CA), mouse anti-PSD-95 (1:1,000; Upstate/CHEMICON), or monoclonal mouse anti-β-actin (1:10,000; Santa Cruz Biotechnology, Inc., Santa Cruz, CA). *N*-cadherin and PSD-95 were used as loading controls and markers for crude membrane fraction, whereas β-actin was used as a loading control for total soluble fraction and cytosolic fraction. The proteins were detected with anti-rabbit or anti-mouse secondary antibody and visualized using the chemiluminescence reagents provided with the ECL kit (Amersham Pharmacia Biotech, Piscataway, NJ) and exposure to film. The intensity of blots was quantified with densitometry. The blot density from naïve animals was set as 100%.

### Subcellular fractionation of proteins

After tissues were homogenized in lysis buffer (10 mM Tris-HCl, 250 mM sucrose, 5 mM MgCl_2_, 2 mM EGTA, 1 mM phenylmethylsulfonyl fluoride, 2 mM sodium orthovanadate, 1 mM NaF, 1 mM leupeptin, 2 mM pepstatin A, 1 mM dithiothreitol), the crude homogenates were centrifuged at 4°C for 15 min at 900 *g*. The supernatant was collected, and the pellet (nuclei and debris fraction) discarded. After the measurement of protein concentration, 20% of the supernatant was removed and considered to be the total soluble fraction. The remaining supernatant (80%) was centrifuged at 37,000 *g *for 1 h at 4°C. The supernatant was considered to be the crude cytosolic fraction, and the pellet, the crude plasma membrane fraction. The pellet was dissolved in buffer (10 mM Tris-HCl, pH 7.4, 1.5% SDS, and 0.1% Triton X-100). Protein concentrations of the three fractions were measured, and samples were prepared for Western blotting, as described above.

### Immunocytochemistry

The animals were deeply anesthetized with isoflurane and perfused with 4% paraformaldehyde in phosphate buffer (0.1 M, pH 7.4) at 2 or 24 h after saline or CFA injection. Naïve rats were used as controls. The L_4–5 _spinal cord segments were harvested, post-fixed in the same fixative solution for 2–4 h, cryoprotected by immersion in 30% sucrose overnight at 4°C, and frozen-sectioned at 25 μm. Every fourth section was collected (at least 30 sections/rat). The sections were blocked for 1 h at 37°C in 0.01 M phosphate-buffered saline (PBS) containing 10% normal goat serum plus 0.3% Triton X-100. Half of the sections (approximately 15 sections/rat) were incubated in primary rabbit antibody for GluR1 (1:500; Upstate/CHEMICON) and the remaining sections in primary mouse antibody for GluR2 (1:1,000; Upstate/CHEMICON) for 48 h at 4°C. The sections were finally incubated in biotinylated goat anti-rabbit or anti-mouse IgG (1:200; Vector Laboratories, Burlingame, CA) for 1 h at 37°C followed by avidin-biotin-peroxidase complex (1:100; Vector) for 1 h at 37°C. The immune reaction product was visualized by catalysis of 3,3-diaminobenzidine by horseradish peroxidase in the presence of 0.01% H_2_O_2_.

Immunostained spinal cord sections were quantified with an Olympus microscope (Olympus, Tokyo, Japan) linked to a Toshiba 3CCD camera (Toshiba, Japan) and I-Cube computer image analysis system (I-Cube, Cambridge, MA). The entire superficial dorsal horn was delimited to quantify the optical density of GluR1 and GluR2 immunoreactivity. Five spinal cord sections randomly selected from a total of 15 sections from each animal were analyzed, and the relative optical densities of five sections were averaged to provide a mean for each animal.

### Statistical Analysis

The results from the behavioral tests, Western blotting, and immunocytochemistry were statistically analyzed with a one-way or two-way analysis of variance (ANOVA). Data are presented as mean ± SEM. When ANOVA showed significant difference, pairwise comparisons between means were tested by the post hoc Tukey method. Significance was set at *p *< 0.05. The statistical software package SigmaStat (Systat, Port Richard, CA) was used to perform all statistical analyses.

## Results

### Effects of intrathecal selective and nonselective AMPAR antagonists on CFA-induced mechanical and thermal hypersensitivities

**Figure 1:**
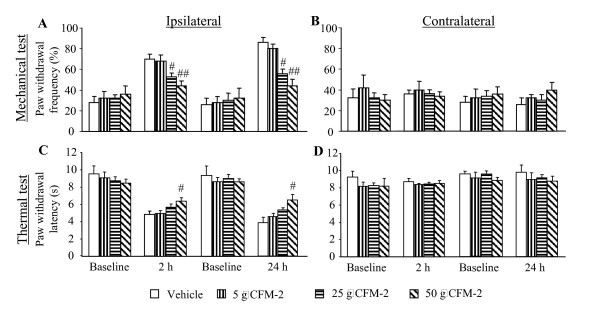
**Effect of intrathecal injection of CFM-2 (5, 25, and 50 μg) on the paw withdrawal responses to mechanical (A and B) and thermal (C and D) stimuli on the ipsilateral (A and C) and contralateral (B and D) sides at 2 and 24 h after intraplantar CFA injection**. Data are presented as mean ± SEM. *n *= 6 rats/dose/test, except *n *= 7 for the vehicle-treated group. # *p *< 0.05, ## *p *< 0.01 *vs *the corresponding vehicle-treated group.

The effect of intrathecal administration of CFM-2 on mechanical and thermal hypersensitivities in animals at 2 h or 24 h after CFA injection was first examined. Consistent with previous studies [17], CFA injection produced a significant increase in paw withdrawal frequency in response to mechanical stimulation (indicating mechanical hypersensitivity; Fig. 1A) and a remarkable decrease in paw withdrawal latency in response to thermal stimulation (indicating thermal hypersensitivity; Fig. 1C) on the ipsilateral side at both 2 h (*n *= 14, 7/test) and 24 h (*n *= 14, 7/test) post-CFA injection in the vehicle group. Intrathecal administration of CFM-2 dose-dependently attenuated CFA-induced mechanical hypersensitivity at 2 h and 24 h post-CFA injection (Fig. 1A). Compared with the corresponding vehicle-treated groups, the 25-μg dose of CFM-2 reduced paw withdrawal frequencies by 26% (*p *< 0.05) and 35% (*p *< 0.01) at 2 h and 24 h (*n *= 6/time point) post-CFA injection, respectively, and the 50-μg dose reduced paw withdrawal frequencies by 37% (*p *< 0.05) and 49% (*p *< 0.01) at these two time points (*n *= 6/time point). Paw withdrawal frequencies after administration of 5 μg of CFM-2 at 2 h (*n *= 6) and 24 h (*n *= 6) post-CFA injection were not significantly different from those of the vehicle group at those time points (*p *> 0.05). Interestingly, only the highest CFM-2 dose (50 μg) significantly reduced the CFA-induced thermal hypersensitivity (Fig. 1C). Compared with vehicle-treated rats, paw withdrawal latencies were increased by 31% at 2 h post-CFA injection (*n *= 6; *p *< 0.05) and by 70% at 24 h post-CFA injection (*n *= 6; *p *< 0.05). Neither the vehicle nor any of the three CFM-2 doses significantly altered basal paw withdrawal responses to mechanical and thermal stimuli applied to the contralateral hind paw at 2 h or 24 h post-CFA injection (Fig. 1B,D). Likewise, none of the doses of CFM-2 significantly affected basal paw withdrawal responses to mechanical and thermal stimuli in naïve rats (data not shown).

**Figure 2:**
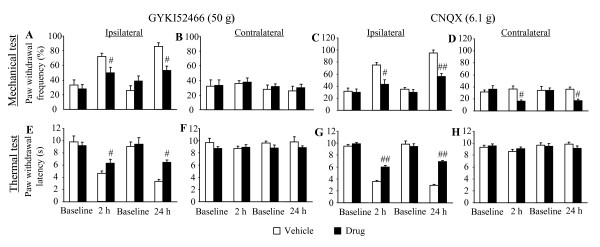
**Effects of intrathecal injection of GYKI 52466 (50 μg, *n *= 6/test/time point, A, B, E, and F) and CNQX (6.1 μg, *n *= 5/test/time point, C, D, G, and H) on the paw withdrawal responses to mechanical (A–D) and thermal (E–H) stimuli on the ipsilateral (A, C, E, and G) and contralateral (B, D, F, and H) sides at 2 and 24 h after intraplantar CFA injection**. Data are presented as mean ± SEM. # *p *< 0.05, ## *p *< 0.01 *vs *the corresponding vehicle-treated group.

To further confirm the effect of selective non-competitive AMPAR antagonists on persistent inflammatory pain, we intrathecally injected another selective non-competitive AMPAR antagonist, GYKI 52466 [15], at 2 h and 24 h post-CFA injection. A 50-μg injection of GYKI 52466 (*n *= 6/test/time point) attenuated both CFA-induced mechanical and thermal hypersensitivities on the ipsilateral hind paw (Fig. 2A,E). Paw withdrawal frequencies were 31% and 38% lower than those of the vehicle-treated group at 2 h (*p *< 0.05) and 24 h (*p *< 0.05) post-CFA injection, respectively (Fig. 2A). Paw withdrawal latencies were 38% and 92.5% higher than those of the vehicle-treated group at 2 h (*p *< 0.05) and 24 h (*p *< 0.05) post-CFA injection, respectively (Fig. 2E). Similar to CFM-2, 50 μg GYKI 52466 had no effect on paw withdrawal responses to thermal or mechanical stimuli applied to the contralateral hind paw at either time point (Fig. 2B,F) or in naïve rats (data not shown).

CNQX is a non-selective competitive AMPAR antagonist. Compared with the responses of the vehicle-treated group (*n *= 20; 5/test/time point), intrathecal CNQX (6.1 μg) significantly reduced CFA-induced mechanical and thermal hypersensitivities at 2 h (*p *< 0.01) and 24 h (*p *< 0.01) after CFA injection (Fig. 2C,G). However, the same dose of CNQX also significantly reduced basal paw withdrawal responses to mechanical stimulation on the contralateral hind paw at both time points (both *p *< 0.05; Fig. 2D), although it had no effect on basal paw withdrawal responses to thermal stimulation on the contralateral hind paw after CFA injection (Fig. 2H).

### Effects of intrathecal selective and nonselective AMPAR antagonists on locomotor functions

To exclude the possibility that the behavioral effects described above were due to impaired motor functions caused by these antagonists, we then examined the effects of CFM-2, GYKI 52466, and CNQX on locomotor functions in naive animals. As indicated in Table 1, at the doses used, none of the antagonists significantly altered placing, grasping, or righting reflexes. In addition, no significant differences were observed in the rats' general behaviors (such as walking and running) between the vehicle-treated and the antagonist-treated groups.

**Table 1:** Effect of selective and non-selective AMPA receptor antagonists on locomotor functions

Treated group	Placing	Grasping	Righting
Saline	5 (0)	5 (0)	5 (0)
DMSO (10%)	5 (0)	5 (0)	5 (0)
CNQX (6.1 μg)	5 (0)	5 (0)	5 (0)
CFM-2 (50 μg)	5 (0)	4.8 (0.45)	5 (0)
GYKI 52466 (50 μg)	4.6 (0.25)	5 (0)	5 (0)

Mean (SE), n = 5, 5 trials

### Effect of CFA-induced peripheral inflammation on expression and distribution of total GluR1 and GluR2 proteins in dorsal horn

**Figure 3:**
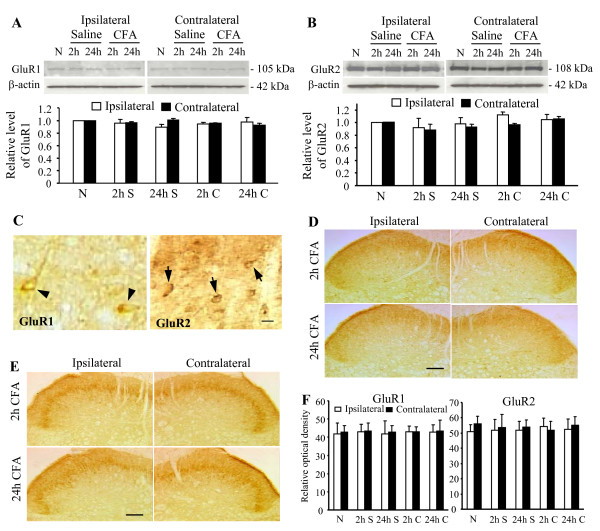
**Expression and distribution of total GluR1 and GluR2 in spinal cord dorsal horn**. A and B: Top: representative Western blots showing GluR1 protein (A) and GluR2 protein (B) in total soluble fraction derived from the ipsilateral and contralateral L_4–5 _dorsal horns of naïve rats (*n *= 4) and the rats at 2 and 24 h post-saline (S; *n *= 4/time point) or post-CFA injection (C; *n *= 4/time point). Bottom: statistical summary of the densitometric analysis expressed relative to the corresponding loading control (β-actin). C: Representative photographs showing the distribution of GluR1 immunoreactive cells (arrow heads) in lamina V and of GluR2 immunoreactive cells (arrows) in laminae II-III. Scale bar: 20 μm. D and E: Representative photographs showing distribution of GluR1 (D) and GluR2 (E) immunoreactivity in the ipsilateral and contralateral L_4 _dorsal horns at 2 and 24 h post-CFA injection. Scale bar: 200 μm. F: Statistical summary of the optical density of GluR1 and GluR2 immunoreactivity in the ipsilateral and contralateral L_4–5 _dorsal horns at 2 and 24 h post-saline (n = 3/time point) or post-CFA (n = 3/time point) injection. N: naïve (n = 3). Data are presented as mean ± SEM.

To further define the involvement of spinal cord AMPARs in persistent inflammatory pain, we examined whether CFA-induced inflammation produced the changes in expression of total GluR1 and GluR2 proteins in total soluble fraction derived from dorsal horn. In the ipsilateral and contralateral L_4–5 _dorsal horn, neither total GluR1 nor total GluR2 protein level was significantly different from that of naïve animals (*n *= 4) at 2 and 24 h post-saline (*n *= 4/time point) and post-CFA (*n *= 4/time point) injection (*p *> 0.05; Fig. 3A, B).

We next examined whether peripheral inflammatory insult affected the distribution of total GluR1 and GluR2 immunoreactivities in the spinal dorsal horn at 2 and 24 h after CFA injection. Consistent with previous studies [12,23,24], GluR1 immunoreactivity was distributed mainly in laminae I and II, whereas GluR2 immunoreactivity was concentrated in inner lamina II and lamina III in naïve rats (*n *= 3). Under high magnification, some GluR1- and GluR2-positive cell bodies were observed in both superficial and deep dorsal horn (Fig. 3C). Neither intraplantar saline nor CFA produced significant changes in the distribution or optical density of GluR1 and GluR2 immunoreactivities in L_4–5 _spinal dorsal horn on the ipsilateral or contralateral side at 2 h (*p *> 0.05) or 24 h (*p *> 0.05) post-saline (*n *= 6, 3/time point) or post-CFA (*n *= 6, 3/time point) injection (Fig. 3D–F).

### Effect of CFA-induced peripheral inflammation on the levels of GluR1 and GluR2 proteins in plasma membrane fraction and cytosolic fraction from dorsal horn

**Figure 4:**
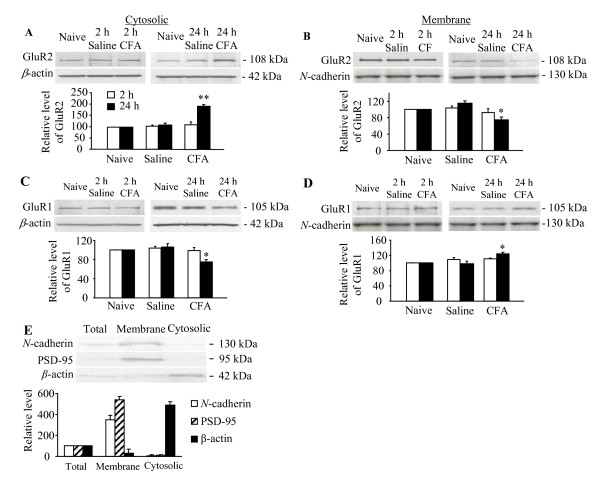
**The relative levels of GluR2 (A and B) and GluR1 (C and D) in the crude cytosolic (A and C) and plasma membrane fractions (B and D) from the ipsilateral L_4–5 _dorsal horn at 2 and 24 h following saline or CFA injection**. Naïve rats (*n *= 4–8) were used as a control group. Data are presented as mean ± SEM. *n *= 4–8 rats/treatment. * *p *< 0.05, ** *p *< 0.01 *vs *the corresponding naïve group. E: The specificity of the fractionation procedure. Top: representative Western blots showing the expression of *N*-cadherin, PSD-95, and β-actin in total soluble fraction, plasma membrane fraction, and cytosolic fraction from L_4–5 _dorsal horns of naïve rats (*n *= 3). Bottom: statistical summary of the densitometric analysis expressed relative to the corresponding protein levels in the total soluble fraction.

Finally, we examined whether CFA-induced peripheral inflammation affected the subcellular distribution of GluR1 and GluR2 proteins in dorsal horn neurons. Quantification of the protein levels revealed that, compared with the naïve animals (*n *= 4), the amount of GluR2 in the CFA-treated group was 90% higher in the cytosolic fraction (Fig. 4A; *p *< 0.01) and correspondingly 26% lower in the membrane fraction (Fig. 4B; *p *< 0.05) at 24 h post-CFA injection (*n *= 4). Conversely, the amount of GluR1 was decreased by 25% in the cytosolic fraction (Fig. 4C; *p *< 0.05) and correspondingly increased by 23% in the membrane fraction (Fig. 4D; *p *< 0.05) at 24 h post-CFA injection (*n *= 8) compared with the values of the naïve group (*n *= 8). Neither the GluR1 nor the GluR2 levels were significantly changed in either the cytosolic (Fig. 4A, C; *p *> 0.05) or membrane (Fig. 4B, D; *p *> 0.05) fraction at 2 h post-CFA injection (*n *= 4). As expected, no significant differences were observed between the saline-treated and naïve groups in either the cytosolic or membrane fractions at 2 or 24 h post-saline injection (Fig. 4; *n *= 4–8/time point).

To test the specificity of the fractionation procedure, the expression levels of a plasma membrane-specific protein (*N*-cadherin), a postsynaptic density protein (PSD-95), and an intracellular protein (β-actin) were assessed in both crude plasma membrane and cytosolic fractions from naïve rats (*n *= 3). Consistent with previous studies [9,22], *N*-cadherin and PSD-95 were detected at high levels in the crude plasma membrane fraction but were barely detectable in the crude cytosolic fraction (Fig. 4E), whereas β-actin was expressed predominately in the crude cytosolic fraction (Fig. 4E), indicating that the fractionation procedure effectively separated cytosolic proteins from plasma membrane proteins.

## Discussion

Peripheral tissue inflammation leads to persistent hyperalgesia in animals that mimics chronic inflammatory pain states in humans. Understanding the mechanisms that underlie persistent inflammatory pain may produce novel therapeutic strategies for prevention and/or treatment of clinical inflammatory pain. Although evidence documented over the last 30 years suggests that AMPARs might be involved in persistent inflammatory pain, the exact role of spinal cord AMPARs in this disorder is unclear. Our study provides pharmacologic evidence that blockade of spinal cord AMPARs produces marked reversal of mechanical and thermal hypersensitivities during the development and maintenance phases of CFA-induced persistent inflammatory pain. In addition, we show that CFA-induced peripheral inflammation leads to GluR2 internalization and GluR1 membrane insertion in dorsal horn neurons, without altering the expression and distribution of total GluR1 and GluR2 proteins in dorsal horn. These findings suggest that spinal cord AMPARs might play a critical role in the central mechanism of persistent inflammatory pain.

The therapeutic potential of AMPAR antagonists in persistent pain has been largely overlooked because it is generally thought that AMPARs are involved primarily in fast synaptic transmission and acute spinal processing of nociceptive and non-nociceptive inputs in the dorsal horn [4]. For example, some earlier studies showed that intrathecal injection of competitive AMPA/kainate receptor antagonists produced dose-dependent antinociception in the tail flick test and hot plate test [25] in rats. Moreover, systemic administration of a competitive AMPA/kainate receptor antagonist, NBQX, reduced chronic allodynia-like response in spinally injured rats; however, it also reduced muscle tone and caused sedation at the dose studied [8]. In our study, intrathecal CNQX, another competitive AMPA/kainate receptor antagonist, markedly attenuated CFA-induced mechanical and thermal hypersensitivities ipsilaterally, but it also significantly reduced basal paw withdrawal response to mechanical stimulation on the contralateral hind paw after peripheral inflammation. These unwanted side effects associated with AMPA/kainate receptor antagonists may prevent them from providing therapeutic potential in persistent pain.

The present study demonstrates that intrathecally administered CFM-2 and GYKI 52466, two highly selective non-competitive AMPAR antagonists [13-15], significantly reverse CFA-induced mechanical and thermal hypersensitivities on the ipsilateral side, without affecting basal nociceptive responses on the contralateral side. Neither CFM-2 nor GYKI 52466 at the doses used affected normal nociceptive responses or locomotor function in naïve animals. These data indicate that CFM-2 and GYKI 52466 at the doses used affect peripheral inflammation-induced behavioral reflex sensitization rather than basal behavioral reflex, suggesting a role of spinal cord AMPARs in mediating the sensitized nociceptive transmission that occurs under persistent inflammatory pain conditions.

Among the four AMPAR subunits, GluR1 and GluR2 are the most abundant in dorsal horn, particularly in the superficial dorsal horn [24,26], where they are highly concentrated on the postsynaptic neuronal membranes [27,28]. We found that CFA injection did not affect the distribution or optical density of GluR1 and GluR2 immunoreactivity in dorsal horn. This finding is inconsistent with a previous report that showed that CFA-induced peripheral inflammation increased the density of GluR1 and GluR2 immunoreactivity in dorsal horn [12]. The reason for the discrepancy between the previous and present results is unclear but might be related to different primary antibody and immunostaining duration. Quantitative Western blot analyses from the present study and those of others [29,30] further demonstrated that the expression levels of total GluR1 and GluR2 proteins were not significantly changed in dorsal horn under CFA-induced inflammatory pain conditions. It is very likely that peripheral inflammatory insult does not alter translation of GluR1 and GluR2 genes and/or degradation of their proteins in dorsal horn neurons.

One important observation that we made is that peripheral persistent inflammation produces distinct changes in the levels of GluR1 and GluR2 in plasma membrane and cytosolic fractions from dorsal horn. Consistent with a recent study [11], the present study showed that CFA injection produced GluR1 membrane insertion in dorsal horn neurons at 24 h post-CFA injection. We also found that GluR2 was markedly internalized in dorsal horn neurons at 24 h post-CFA injection. Moreover, the number of internalized GluR2 subunits is likely greater than the number of GluR1 subunits inserted in the membrane. Electrophysiological studies from our laboratory and those of others demonstrated that Ca^2+^-permeable AMPARs were significantly increased in the superficial dorsal horn neurons at 24 and 72 h post-CFA injection [31,32]. Because only GluR2 determines the properties of synaptic AMPAR function, particularly Ca^2+ ^permeability [3], GluR2 internalization might be a major player in AMPAR subunit trafficking in dorsal horn neurons during the maintenance of persistent inflammatory pain syndromes.

An unexpected observation in the current study was that CFA injection did not produce a marked change in the levels of GluR1 and GluR2 in subcellular fractions from dorsal horn at 2 h post-CFA injection. The reason for the discrepancy in AMPAR trafficking at 2 h and 24 h post-CFA injection is unclear, but it may be related to the limited sensitivity of Western blotting. It is possible that GluR1 membrane insertion and GluR2 internalization in dorsal horn neurons are too minimal at 2 h post-CFA injection to be detected by this approach. In addition, synaptic AMPAR exchange for intracellular receptors in dorsal horn neurons may have a long timescale because synaptic AMPAR trafficking events *in vitro *and in intact brains have a slow rate time constant of ~15–18 h [33,34].

The molecular mechanisms by which GluR1 is inserted into plasma membrane and GluR2 is internalized into cytoplasm in dorsal horn neurons during the maintenance phase of persistent inflammatory pain are unclear, but they might be related to inflammation-induced spinal cord GluR1 and GluR2 phosphorylation. Protein kinase A phosphorylation of GluR1 Ser^845 ^promotes the surface-expression of GluR1 in cultured hippocampal neurons during activity-dependent long-term potentiation [35] and might also be responsible for spinal GluR1 membrane insertion *in vivo *following CFA-induced inflammation. Similarly, Protein kinase C phosphorylation of GluR2 at Ser^880 ^disrupts GluR2 binding to its synaptic anchoring protein ABP/GRIP and promotes GluR2 internalization in cultured hippocampal neurons [36,37]. Such a mechanism might also contribute to CFA-induced internalization of GluR2 subunits *in vivo*. It will be very important to further elucidate these mechanisms to fully understand the role of spinal AMPAR trafficking in the spinal central sensitization that underlies persistent inflammatory pain.

In conclusion, we demonstrated that selective blockade of spinal AMPAR activity significantly reduces mechanical and thermal hypersensitivities during the development and maintenance of persistent inflammatory pain. We also showed that GluR2 is likely transported away from the plasma membrane and GluR1 inserted into plasma membrane of dorsal horn neurons during the maintenance (but not the development) of persistent inflammatory pain. These findings imply that spinal cord AMPARs are involved in the central mechanism of persistent inflammatory pain.

## Abbreviations

AMPA: α-amino-3-hydroxy-5-methyl-4-isoxazolepropionic acid; AMPAR: AMPA receptor; ANOVA: analysis of variance; CFA: complete Freund's adjuvant; DMSO: dimethylsulfoxide; GluR1: glutamate receptor subunit 1; GluR2: glutamate receptor subunit 2; PBS: phosphate-buffered saline.

## Competing interests

The authors declare that they have no competing interests.

## Authors' contributions

JSP carried out all behavioral experiments and most biochemical experiments. MY, XG, JTX, and MHS assisted with immunocytochemical experiments and the data analysis. YG and SNR participated in the data interpretation and critical review manuscript. YXT contributed to the design of the study, some biochemical experiments, the data analysis and interpretation, and drafting and critical review the manuscript.
